# ELABELA ameliorates hypoxic/ischemic-induced bone mesenchymal stem cell apoptosis via alleviation of mitochondrial dysfunction and activation of PI3K/AKT and ERK1/2 pathways

**DOI:** 10.1186/s13287-020-02063-1

**Published:** 2020-12-14

**Authors:** Jiaying Fu, Xuxiang Chen, Xin Liu, Daishi Xu, Huan Yang, Chaotao Zeng, Huibao Long, Changqing Zhou, Haidong Wu, Guanghui Zheng, Hao Wu, Wuming Wang, Tong Wang

**Affiliations:** 1 Department of Emergency, the Eighth Affiliated Hospital of Sun Yat-sen University, Shenzhen, 518033 Guangdong People’s Republic of China; 2 Department of Emergency, the Sun Yat-sen Memorial Hospital of Sun Yat-sen University, Guangzhou, 510120 Guangdong People’s Republic of China

**Keywords:** ELABELA, Mesenchymal stem cells, Putative receptor protein related to the angiotensin receptor AT1 endogenous ligand, Hypoxic/ischemic, Apoptosis

## Abstract

**Background:**

Mesenchymal stem cells (MSCs) have exerted their brilliant potential to promote heart repair following myocardial infarction. However, low survival rate of MSCs after transplantation due to harsh conditions with hypoxic and ischemic stress limits their therapeutic efficiency in treating cardiac dysfunction. ELABELA (ELA) serves as a peptide hormone which has been proved to facilitate cell growth, survival, and pluripotency in human embryonic stem cells. Although ELA works as an endogenous ligand of a G protein-coupled receptor APJ (Apelin receptor, APLNR), whether APJ is an essential signal for the function of ELA remains elusive. The effect of ELA on apoptosis of MSCs is still vague.

**Objective:**

We studied the role of ELABELA (ELA) treatment on the anti-apoptosis of MSCs in hypoxic/ischemic (H/I) conditions which mimic the impaired myocardial microenvironment and explored the possible mechanisms in vitro.

**Methods:**

MSCs were obtained from donated rats weighing between 80~120 g. MSCs were exposed to serum-free and hypoxic (1% O_2_) environments for 24 h, which mimics hypoxic/ischemic damage in vivo, using serum-containing normoxic conditions (20% O_2_) as a negative control. MSCs that were exposed to H/I injury with ELA processing were treated by 5 μM of ELA. Cell viability and apoptosis of MSCs were evaluated by CCK8 and flow cytometry, respectively. Mitochondrial function of MSCs was also assessed according to mitochondrial membrane potential (MMP) and ATP content. The protein expression of key kinases of the PI3K/AKT and ERK1/2 signaling pathways involving t-AKT, p-AKT, t-ERK1/2, and p-ERK1/2, as well as apoptosis-related protein expression of Bcl-2, Bax, and cleaved Caspase 3, were monitored by Western blot.

**Results:**

We found that ELA treatment of H/I-induced MSCs improved overall cell viability, enhanced Bcl/Bax expression, and decreased Caspase 3 activity. ELA inhibited H/I-induced mitochondrial dysfunction by increasing ATP concentration and suppressing the loss of mitochondrial transmembrane potential. However, this anti-apoptotic property of ELA was restrained in APJ-silenced MSCs. Additionally, ELA treatment induced the phosphorylation of AKT and ERK, while the blockade of PI3K/AKT and ERK1/2 pathways with respective inhibitors, LY294002 and U0126, suppressed the action of ELA.

**Conclusion:**

ELA positively affected on the survival of MSCs and exhibited anti-apoptotic characteristics when exposed to hypoxic/ischemic condition in vitro. Also, the function of ELA was correlated with the APJ receptor, reduced mitochondrial damage, and activation of the PI3K/AKT and ERK1/2 signal axes.

## Background

Mesenchymal stem cells (MSCs), as a promising cell resource, have emerged to be a desirable method for stem cell-based therapy [1, 2]. Over the past few years, a large quantity of animal experiments and clinical trials have verified the reparative effects of MSC implantation after myocardial infarction [3, 4]. Alone, the adult heart is incapable of efficient regeneration for repairing injured myocardium, which poses a dilemma for clinicians worldwide. Fortunately, MSC transplantation has been shown to improve cardiac function via anti-inflammation, anti-fibrotic, and regenerative properties [5, 6]. MSCs possess characteristics involving self-renewal capacity, multi-direction differentiation, and paracrine signaling under appropriate conditions. However, the poor survival and apoptosis of MSCs still remain unsolved within local ischemic/hypoxic environments [7]. Ongoing studies are currently exploring the molecular mechanism behind the ischemic/hypoxic microenvironment on MSC apoptosis [8].

Apoptosis is generally considered as a vital process to maintain the homeostasis of both tissues and cells, which is regulated by sophisticated apoptotic signals [9]. A mass of evidence suggests that PI3K/AKT and ERK1/2 signaling pathways are related to cell proliferation and apoptosis [10–12]. Ascending expression of AKT phosphorylation and ERK1/2 phosphorylation seems to be pivotal anti-apoptotic signals [13, 14]. Moreover, Caspase 3 acts as the key executor of apoptosis [15]. Under normal circumstances, Caspase 3 exits as an inactive precursor, while Caspase 3 can be activated by apoptotic signals and converted into cleaved Caspase 3 [16]. Being members of Bcl-2 family proteins, Bcl-2 works as an anti-apoptotic protein and Bax as a pro-apoptotic protein [17]. The heterodimer of Bcl-2 and Bax is formed to regulate cell apoptosis and plays an important role in mitochondrial membrane permeability [18, 19]. Additionally, the mitochondria are the main locations where cells produce adenosine triphosphate (ATP), which is closely related to the process of apoptosis [20]. Ischemic and hypoxic factors are two leading causes of mitochondrial dysfunction involving excessive accumulation of reactive oxygen species, mitochondrial transmembrane potential depolarization, and decreased ATP production [21]. Therefore, it is of great significance to figure out feasible cell processing to promote the resistance of MSCs to oxidative stress.

ELABELA (ELA), a peptide hormone, is secreted by the placenta [22]. Apart from the putative receptor protein related to the angiotensin receptor AT1 endogenous ligand (Apelin), ELA has been regarded as the second endogenous ligand for a kind of orphaned G protein-coupled receptor called APJ (Apelin receptor, APLNR) [23]. The ELA/APJ system is relevant to multiple biological functions, such as mesodermal differentiation, cardiogenesis, and treatment targets for heart diseases [24–26]. Ho et al. discovered that ELA protected human embryonic stem cells against cellular stress and enhanced their self-renewal ability through PI3K/AKT pathway [27]. Our previous discoveries showed that Apelin could reinforce the survival of MSCs during the hypoxic-ischemic conditions [28]; therefore, it was of great interest to us whether ELA has a positive effect on MSCs similar to Apelin.

In the present study, the influence of ELA on the anti-apoptosis of MSCs during ischemic/hypoxic injury and the underlying mechanisms in vitro were investigated.

## Materials and methods

### Ethics statement

Sprague-Dawley rats weighting between 80~120 g were purchased from the Animal Experimental Centre of Guangzhou University of Chinese Medicine. All relevant animal experiments and tests were approved by the Animal Ethics Committee of Sun Yat-sen University (2019-057-01).

### Chemicals

An ELA peptide of 32 amino acids (sequence: QRPVNLTMRRKLRKHNCLQRRCMPLHSRVPFP) was purchased from GL Biochem Shanghai Ltd (China). The ELA powder was at 96.08% purity and stored at − 20 °C. The ELA was dissolved and diluted with PBS to 5 μM and then sterilized with 0.22 μm filters before utilization.

### Cell isolation and culture

MSCs were isolated from the femurs and tibias of Sprague-Dawley rats. Under sterile conditions, the marrow cavities were rinsed with PBS buffer containing 1% penicillin/streptomycin solution. Cells were then cultured in the standard medium, low-glucose Dulbecco’s modified Eagle’s medium (GIBCO, USA) supplemented with 10% fetal bovine serum (GIBCO, USA) and 1% penicillin/streptomycin solution (100 U/mL, HyClone, USA). Three days later, the culture medium was replaced for the sake of removing those non-adherent cells. When reached approximately 80~90% confluence, adherent cells were passaged at a ratio of 1:2. MSCs at passage 3 were used for the following experiments. Phenotypic cell surface markers of MSCs including CD34, CD44, and CD29 were detected by fluorescence-activated cell sorting for characterization, as previously reported [28, 29]. The third-passage MSCs were positive for both CD29 and CD44 but were negative for CD34, being consistent with the results of other researchers [30, 31].

### APJ silencing by RNA interference

MSCs were incubated in culture medium without the penicillin/streptomycin solution. The small interfering RNAs (siRNA) targeting APJ (siRNA-APJ, sequence: GCCTCAGCTTTGACCGATA) and scramble negative control of siRNA were synthesized by RiboBio Co. (Guangzhou, China). According to the manufacturer’s instructions, cells were transfected with siRNA-APJ or siRNA-APJ NC by using Lipofectamine RNAiMax Reagent (ThermoFisher, USA).

### Hypoxia/ischemia (H/I) model and treatments of MSCs

A total of nine groups were established with different treatments: (1) control group—negative control with no special treatment; (2) siAPJ group—MSCs transfected with siRNA-APJ; (3) siAPJ NC group—MSCs transfected with siRNA-APJ NC; (4) H/I group—MSCs incubated with serum-deprived medium in a hypoxia incubator chamber (STEMCELL, Canada) containing 1% O_2_, 94% N_2_, and 5% CO_2_ for 24 h [28, 29]; (5) ELA group—MSCs cultivated in serum-deprived medium with 5 μM ELA in a hypoxia incubator chamber for 24 h; (6) siAPJ + ELA group—MSCs transfected with siRNA-APJ before treatment in the ELA group; (7) siAPJ NC + ELA group—MSCs transfected with siRNA-APJ NC before treatment in the ELA group; (8) LY294002 + ELA group—MSCs pretreated with 50 μM LY294002 (PI3K/AKT pathway inhibitor; Med Chem Express, USA) for 2 h prior to ELA; and (9) U0126 + ELA group—MSCs pretreated with 10 μM U0126 (ERK pathway inhibitor; Med Chem Express, USA) for 2 h prior to ELA. It should be noted that MSCs in groups 6 through 9 were then handled under the same process as the ELA group after the abovementioned treatment.

### Cell viability assay

A cell counting kit-8 (CCK8; APExBio, USA) was executed to determine the cell viability. After specified processing in each group, 100 μL cell suspension (at a density of 3 × 10^4^) was plated in 96-well plates with the addition of 10 μL CCK8 reagent and was then cultured at 37 °C for 2 h.

In addition, a microplate reader (Thermo Varioskan LUX, USA) was used to measure the absorbance at a wavelength of 450 nm. The percentage of cell viability was subsequently obtained by calculating the mean optical density (OD) in each group.

### Cell apoptosis assay

The Annexin V–FITC/PI apoptosis detection kit (BD, USA) was applied to assess the apoptosis of MSCs according to the standard protocol. After designated treatment, cells were obtained with 0.25% trypsin (without EDTA) and centrifugated at 1000 rpm for 5 min. Subsequently, MSCs were washed twice with cold PBS and resuspended in 100 μL 1× Binding Buffer. Five microliters Annexin V–FITC and 5 μL PI were added and gently blended in each tube. MSCs were then incubated in the dark at room temperature for 15 min before additional 400 μL 1× Binding Buffer was mixed into each tube. At final, cell samples were detected by flow cytometry (BD LSRFortessa, USA) within an hour.

### Mitochondrial membrane potential (MMP) assay

The change of MMP (Δψm) was detected by using the mitochondrial membrane potential assay kit with JC-1 (Beyotime, China) in accordance with the manufacturer’s instruction. Before that, the as-prepared cells were transferred into 6-well plates at a concentration of 1 × 10^6^ per well and were incubated with JC-1 solution in the darkroom at 37 °C for 20 min. JC-1 staining buffer (1×) was prepared and was placed at 4 °C. Then, cells were washed twice with the pre-cooling buffer. JC-1 fluorescence in each group was subsequently monitored with a confocal laser scanning microscope (ZEISS, Germany). It should be noted that JC-1 monomers were investigated at excitation/emission (green signal) = 514/530 nm, while JC-1 aggregates were at excitation/emission (red signal) = 525/590 nm. According to the ratio of red/green fluorescence, Δψm was quantified and was normalized to the fluorescent baseline of the control group.

### Measurement of ATP content

The ATP content was measured using an ATP assay kit (Beyotime, China) following the instructions. Cells seeded in 6-well plates (at a density of 5 × 10^5^ cells per well) were treated with 200 μL ATP lysis buffer and were then centrifuged at 4 °C, 12,000×*g* for 5 min. Then, 20 μL supernatant in each group was transferred to a 96-well Solid White Flat Bottom Polystyrene TC-treated Microplate (Corning, USA) supplemented with 100 μL ATP detection solution. Luminance (RLU) of the mixtures was observed via measurement of a microplate reader (Thermo Varioskan LUX, USA).

### Western blot

The as-prepared cells in 6-well plates were washed twice with PBS before lysed in RIPA lysis buffer (Beyotime, China) incorporated with protease and phosphatase inhibitor cocktail (CWBIO, China) for 30 min on ice. Supernatant in each group was harvested after centrifugation at 12,000×*g* for 15 min, the protein concentration of which was determined through a bicinchoninic acid (BCA) assay kit (CWBIO, China). Mixed with loading buffer, the protein samples were then boiled at 100 °C for 10 min. Equal-sized protein samples were separated by 12% SDS-PAGE and were then transferred to a 0.2-μm polyvinylidene fluoride (PVDF) membrane (Millipore, USA). Then, the membranes were incubated at room temperature in a blocking solution (5% skim milk in 1× TBST) for 1 h. The membrane was subsequently incubated with anti-rabbit primary antibodies at 4 °C overnight: AKT (1:1000; # 4691; Cell Signaling Technology, USA), phospho-Akt (Ser473) (1:2000; # 4060; Cell Signaling Technology, USA), p44/42 ERK1/2 (1:1000; # 4695; Cell Signaling Technology, USA), phospho-p44/42 Erk1/2 (1:2000; # 9101; Cell Signaling Technology, USA), Bcl-2 (1:500; # ab32124; Abcam, USA), Bax (1:1000; # 2772; Cell Signaling Technology, USA), Caspase 3 (1:500; # 9662; Cell Signaling Technology, USA), cleaved Caspase 3 (1:500; # 9664; Cell Signaling Technology, USA), APJ (1:600; # 20341-1-AP; Proteintech, USA), and GAPDH (1:1000; # 2118; Cell Signaling Technology, USA). On the second day, 1× TBST was used to wash the membranes three times for 10 min each, and after that, the membranes were incubated with anti-rabbit IgG, HRP-linked antibody (1:2000, Cell Signaling, USA) at room temperature for 1 h. After rinsing three times with 1× TBST for 5 min each, the bands treated with chemiluminescence reagents were detected by the ChemiDoc™ Touch Imaging System (Bio-Rad, USA).

### Statistical analysis

Quantitative data were expressed as mean ± SD. Each experiment was repeated at least three times. Analysis of Student’s *t* test was used for comparison between two groups. One-way analysis of variance (ANOVA) with a Bonferroni post hoc test was applied in the comparison between groups. *P* < 0.05 represents statistical significance.

## Results

### RNA interference efficiency of APJ siRNA

**Fig. 1:**
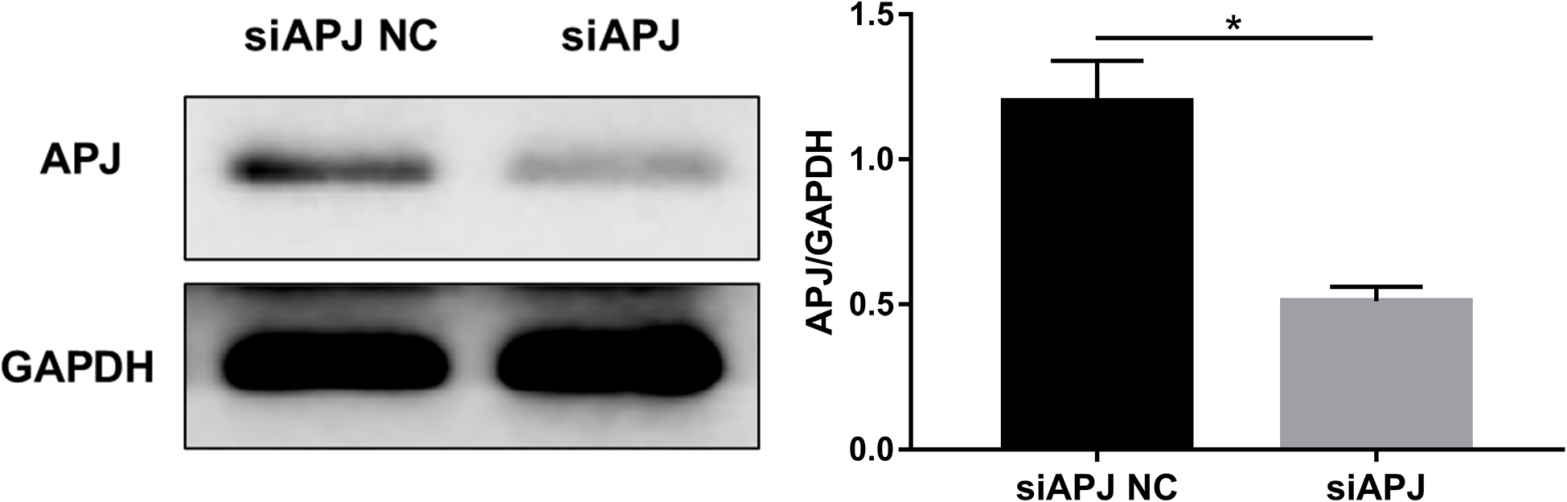
Interference efficiency of siRNA-APJ in MSC detected by immunoblot analysis. The representative image and quantified expression of APJ were shown (*N* = 3). *P < 0.05. MSC, mesenchymal stem cell; siRNA, small interfering RNA

To determine whether APJ was a vital receptor for ELA to regulate cell survival of MSC, APJ gene silencing was carried out. Westerm blot was used to determine the APJ interference efficiency in MSCs. The results showed a decreased expression of APJ in cells pretreated with siRNA-APJ, while there was no knockdown of APJ that could be seen in the siAPJ NC group (*P* < 0.05, Fig. 1).

### ELA enhanced MSC survival under hypoxic/ischemic condition

**Fig. 2:**
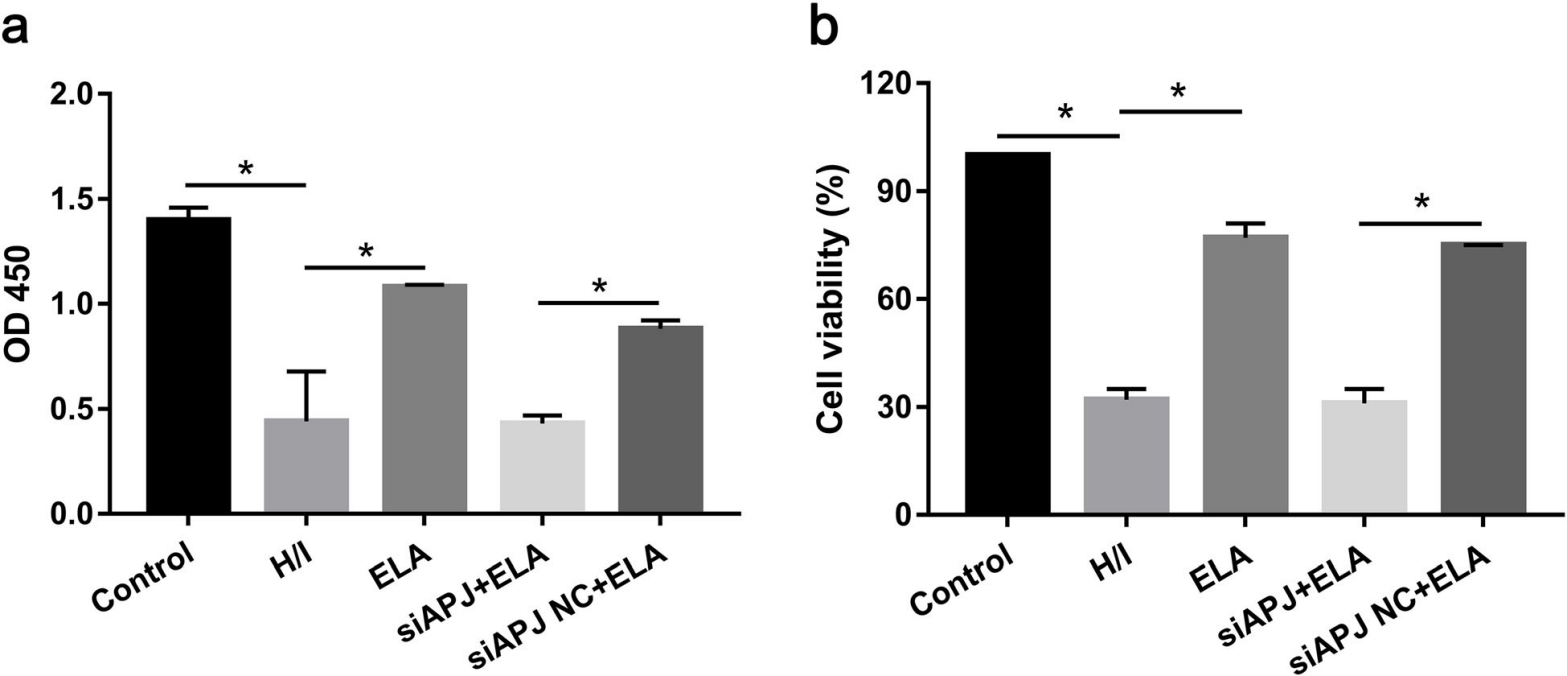
ELA promoted MSC proliferation (**a**) and viability (**b**) under H/I exposure. Cell viability = (OD value in each group − OD value of CCK8 and medium/OD value in control group − OD value of CCK8 and medium) × 100%. All experiments were performed in triplicate. *P < 0.05. ELA, ELABELA; MSC, mesenchymal stem cell; H/I, hypoxic/ischemic; CCK8, cell counting kit-8

After 24-h exposure to the hypoxic/ischemic microenvironment, cell survival of MSCs declined. We demonstrated that ELA treatment promoted cell proliferation and increased the survival rate of MSCs (control group 1.4 ± 0.06 (100%) versus H/I group 0.44 ± 0.24 (31.61 ± 2.65%), *P* < 0.05, Fig. 2a, b). However, when compared to the siAPJ NC + ELA group, the siAPJ + ELA group displayed a lower growth rate (siAPJ NC + ELA group 0.88 ± 0.04 (74.63 ± 0.13%) versus siAPJ + ELA group 0.43 ± 0.04 (30.61 ± 3.90%), *P* < 0.05, Fig. 2a, b). And no distinct difference can be observed between the H/I group and siAPJ + ELA group (H/I group 0.44 ± 0.24 (31.61 ± 2.65%) versus siAPJ + ELA group 0.43 ± 0.04 (30.61 ± 3.9%), *P* > 0.05, Fig. 2a, b), indicating that ELA might not confer protection on MSCs with APJ deficiency.

### ELA protected MSCs from hypoxia/ischemia-induced apoptosis

**Fig. 3:**
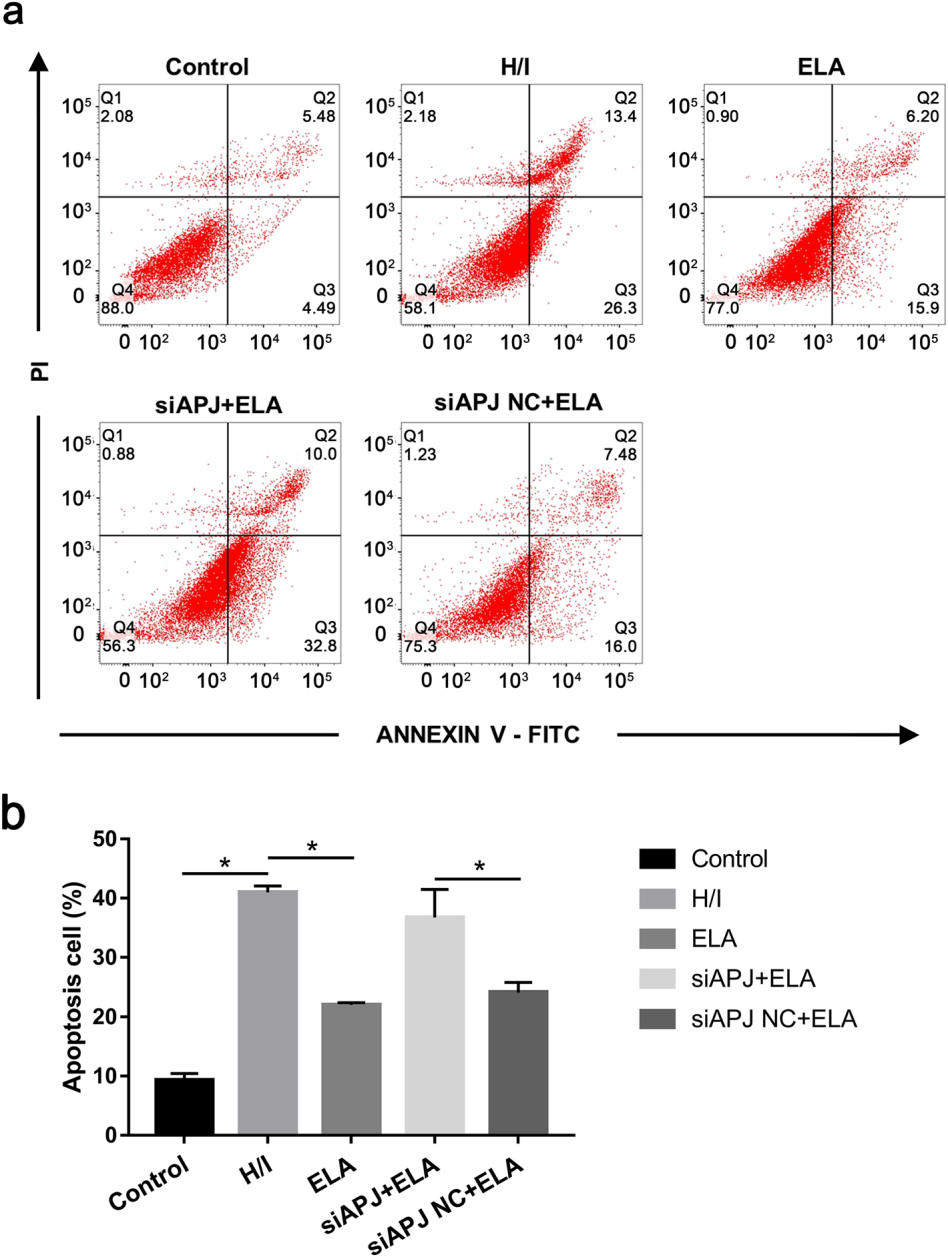
Effect of ELA on H/I-induced MSC apoptosis. **a** MSC apoptosis results obtained from flow cytometry of various experimental groups. **b** Apoptotic cell rates were statistically presented as mean ± SD. Three parallel experiments were conducted independently. *P < 0.05. ELA, ELABELA; MSC, mesenchymal stem cell; H/I, hypoxic/ischemic

Consistent with cell survival, ELA treatment in the ELA group protected MSCs against H/I**-**induced apoptosis (ELA group 22.04 ± 0.37% versus H/I group 40.97 ± 1.1%, *P* < 0.05, Fig. 3a, b). Besides, for the H/I group and siAPJ + ELA group, they presented similar figures (H/I group 40.97 ± 1.1% versus siAPJ + ELA group 36.73 ± 4.74%, *P* > 0.05, Fig. 3a, b). Moreover, cell apoptosis in the siAPJ + ELA group was elevated compared to the siAPJ NC + ELA group (siAPJ + ELA group 36.73 ± 4.74% versus siAPJ NC + ELA group 24.1 ± 1.67%, *P <* 0.05, Fig. 3a, b).

### ELA inhibits mitochondrial dysfunction induced by hypoxic/ischemic injury

In order to assess the inhibition of ELA on H/I-induced mitochondrial dysfunction, we measured the MMP (MMP, Δψm) and ATP production.

**Fig. 4:**
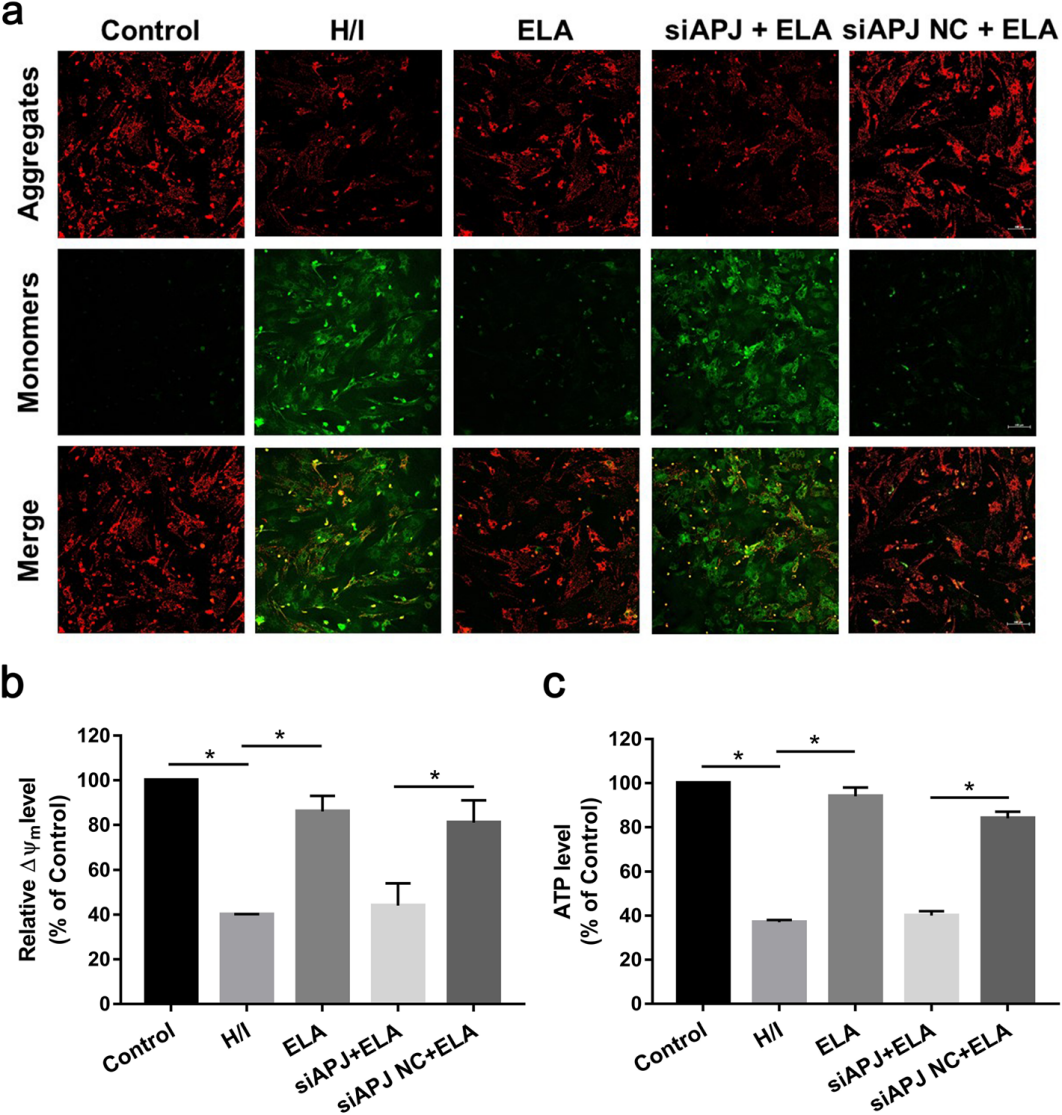
ELA treatment ameliorated H/I-induced mitochondrial dysfunction in MSCs. **a** Representative confocal images of JC-1 staining. Scale bar = 100 μm. **b** Quantitative analysis of mitochondrial membrane potential with JC-1 staining. **c** ATP content was evaluated using a luminescence enzymatic assay. The ATP level in each group was expressed as the bioluminescence intensity that was normalized to the control group (*N* = 3). *P < 0.05. ELA, ELABELA; MSC, mesenchymal stem cell; H/I, hypoxic/ischemic; ATP, adenosine triphosphate

Δψm, as a critical sign of apoptosis occurrence, was detected through JC-1 staining. Under normal conditions, JC-1 is assembled in the mitochondrial matrix and exists in the form of JC-1 aggregates, displaying red fluorescence. In contrast, the Δψm of apoptotic cells are depolarized and JC-1 fails to polymerize in the mitochondrial matrix and exists as JC-1 monomers, displaying green fluorescence. Therefore, Δψm was regarded as the red/green fluorescent ratio. In comparison with the control group, the MMP in the H/I group was collapsed (control group 100% versus H/I group 40.08 ± 0.43%, *P <* 0.05, see Fig. 4a, b). However, ELA treatment suppressed the loss of MMP, which was not observed in the siRNA + ELA group (ELA group 85.63 ± 6.54% versus siAPJ + ELA group 43.79 ± 0.51% versus siAPJ NC + ELA group 81.2 ± 1.4%, Fig. 4a, b). Furthermore, ATP results showed a similar pattern that ELA could motivate ATP production in MSCs under H/I condition (ELA group 94.15 ± 4.28% versus H/I group 36.71 ± 0.83%, *P <* 0.05, Fig. 4c). ELA treatment in MSCs without siRNA-APJ transfection improved the decrease of ATP generation caused by the H/I environment, as compared to the siAPJ + ELA group (siAPJ + ELA group 40.46 ± 1.54% versus siAPJ NC + ELA group 84.1 ± 3.46%, *P <* 0.05, Fig. 4c). Thus, these outcomes demonstrated that ELA regulated the mitochondrial dysfunction induced by H/I via APJ.

### ELA modulates the ratio of Bcl-2 to Bax and Caspase 3 activity in the hypoxic/ischemic microenvironment

**Fig. 5:**
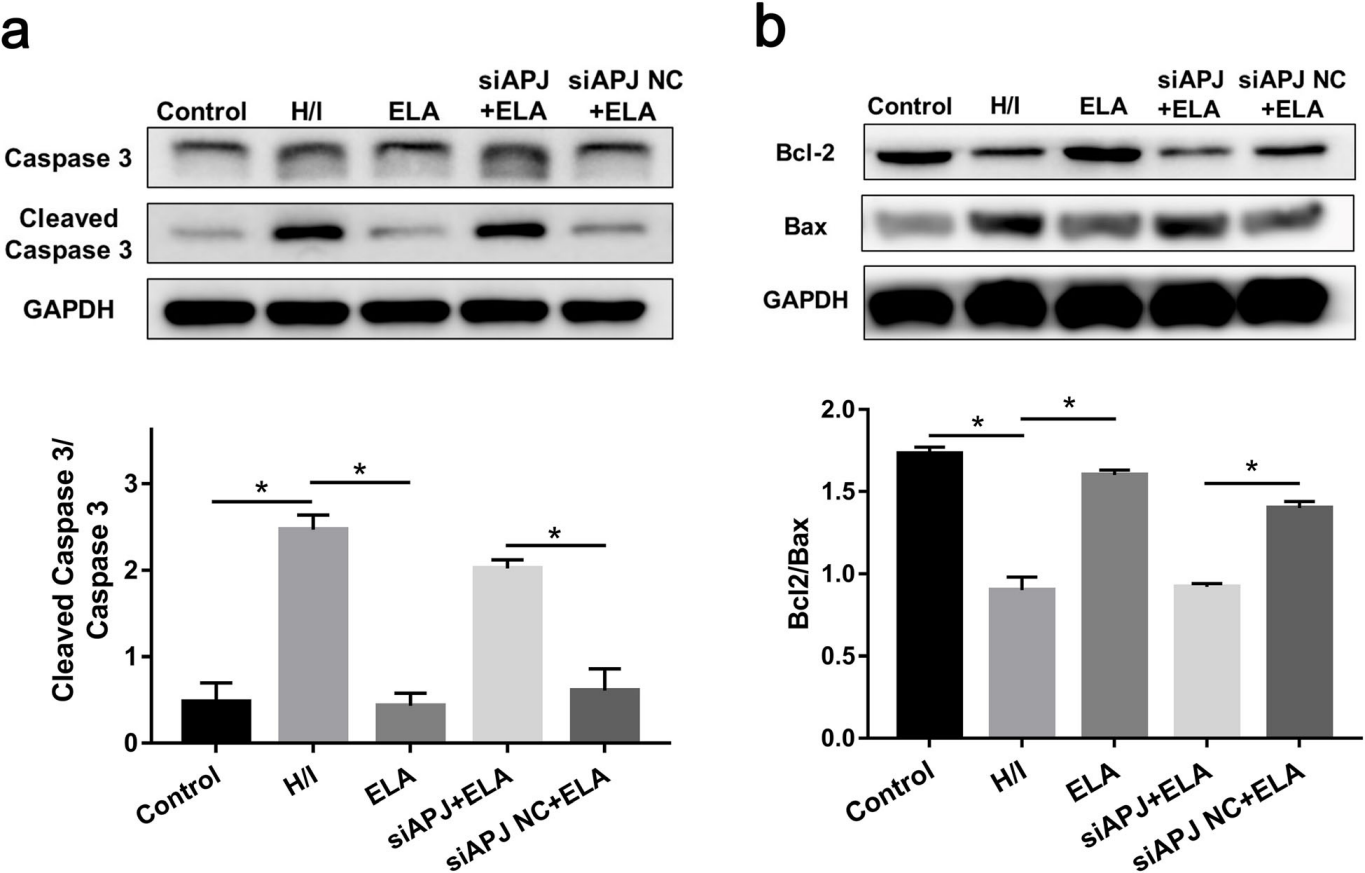
Effects of ELA on Caspase 3 activity and balance of Bcl-2 and Bax expression in MSC under H/I injury. Representative immunoblots and quantitative data were provided. **a** Cleaved Caspase 3/Caspase 3 expression in different experimental groups. **b** The ratio of Bcl-2/Bax in different experimental groups (*N* = 3). *P < 0.05. ELA, ELABELA; MSC, mesenchymal stem cell; H/I, hypoxic/ischemic

Caspase 3 activity was calculated by the ratio of cleaved Caspase 3/Caspase 3. Compared to the control group, cleaved Caspase 3/Caspase 3 expression was remarkably enhanced in the H/I group, while this promotion was reversed under ELA treatment in the ELA group (*P <* 0.05, Fig. 5a). Despite this, Caspase 3 activity remained at a high level in the siAPJ + ELA group (Fig. 5a).

Additionally, the H/I group saw a decline of Bcl-2/Bax ratio as compared to the control group, which suggested that Bax was more dominant during the H/I process (*P <* 0.05, Fig. 5b). However, a dramatic increase of Bcl-2/Bax ratio could be seen in the ELA group, while the ratio of the siAPJ + ELA group still remained low (*P <* 0.05, Fig. 5b). The aforementioned results revealed that ELA ameliorated the apoptosis of MSCs by modulating the Caspase 3 activity and the ratio of Bcl-2/Bax.

### ELA activates ERK1/2 and PI3K/AKT pathways against hypoxic/ischemic injury

**Fig. 6:**
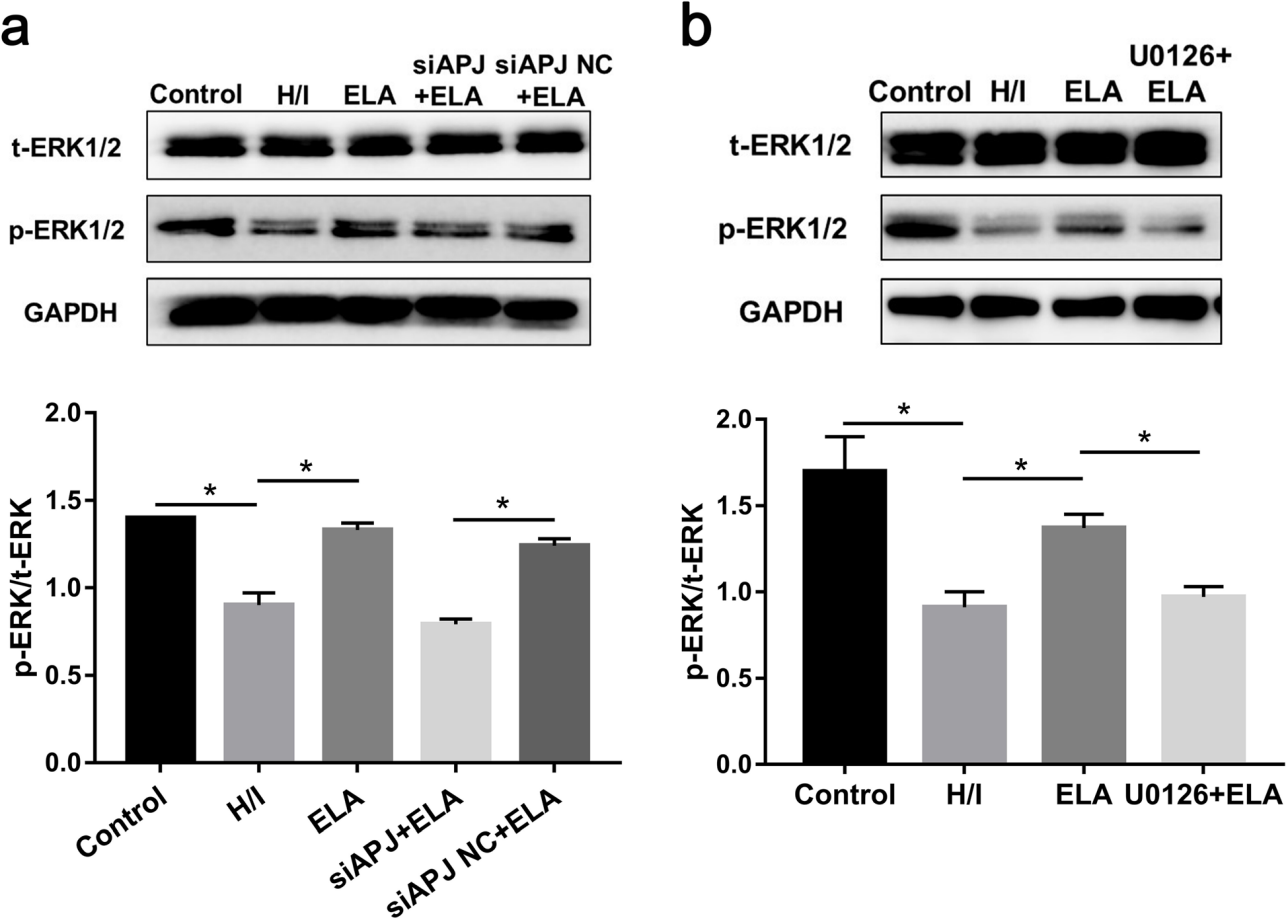
ELA activated the ERK1/2 signaling pathway in reducing the apoptosis of MSCs caused by H/I condition. Representative immunoblots and quantitative data were provided. **a** t-ERK1/2 and p-ERK1/2 expression in separate groups. **b** Utilization of U0126 further verified the role of ELA in activating ERK1/2 phosphorylation (*N* = 3). *P < 0.05. ELA, ELABELA; MSC, mesenchymal stem cell; H/I, hypoxic/ischemic; t-ERK1/2, total ERK1/2; p-ERK1/2, phosphorylated ERK1/2

**Fig. 7:**
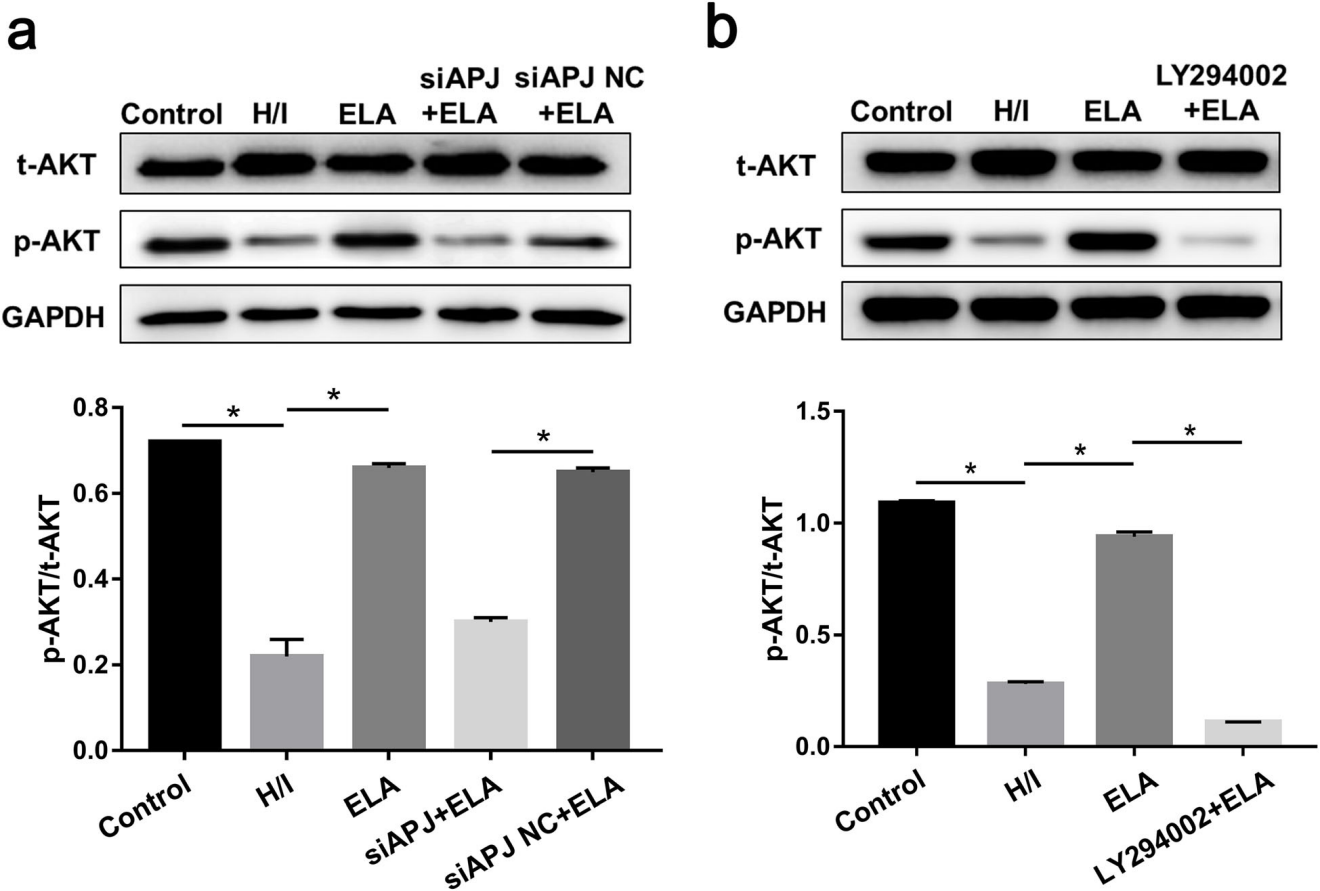
PI3K/AKT signaling pathway was related to ELA effects on MSC anti-apoptosis under H/I injury. Representative immunoblots and quantitative data were provided. **a** t-AKT and p-AKT expression in separate groups. **b** Utilization of LY294002 further identified the role of ELA in activating AKT phosphorylation (*N* = 3). *P < 0.05. ELA, ELABELA; MSC, mesenchymal stem cell; H/I, hypoxic/ischemic; t-AKT, total AKT; p-AKT, phosphorylated AKT

To gain insights into the mechanism behind ELA, key kinases related to both ERK1/2 and PI3K/AKT signaling pathways were detected. The expression of p-ERK1/2 and p-AKT appeared to decline during the H/I process, while the expression showed an increase in the ELA group (*P <* 0.05, Figs. 6a and 7a). Interestingly, ELA-treated MSCs with APJ deficiency demonstrated low expressions of ERK1/2 phosphorylation and AKT phosphorylation (Figs. 6a and 7a).

U0126 was utilized to prove whether ERK1/2 phosphorylation was related to ELA. In a similar way, LY294002 was employed to verify if AKT phosphorylation was regulated by ELA. We found that U0126 evidently abrogated ERK1/2 phosphorylation caused by ELA (Fig. 6b). Likewise, LY294002 reversed AKT phosphorylation activated by ELA during H/I injury (Fig. 7b), suggesting that under H/I conditions, the anti-apoptotic effects of ELA on MSCs could be partially ascribed to the activation of ERK1/2 and PI3K/AKT pathways.

## Discussion

In the present study, ELA treatment exerted an anti-apoptotic action against H/I-induced apoptosis of MSCs. APJ appeared to be a vital receptor for the anti-apoptotic property of ELA on MSCs. ELA-treated MSCs presented improved mitochondrial dysfunction, and the anti-apoptotic signaling pathway was activated by ELA involving PI3K/AKT and ERK1/2.

Continuous ischemic and hypoxic attacks cause irreversible myocardial loss, ventricular remodeling, heart failure, and eventually death [32]. Despite rapid developments in stem cell therapy, low survival rates of transplanted MSCs in the hypoxia/ischemia microenvironment have been an issue that needs to be urgently addressed [33, 34]. In view of the anti-apoptotic effect of ELA on MSCs under hypoxic/ischemic conditions in vitro, ELA treatment appears to be a proper way for prolonging the lifespan of MSCs. Moreover, it is worth further exploring whether ELA-treated MSC transplantation can improve the therapeutic efficiency of myocardial infarction model mice in vivo*.*

Apoptosis is an orderly process of cell death that is regulated by sophisticated signal transduction mechanisms [35]. In this study, MSCs exhibited a higher apoptosis rate in the H/I state. In contrast, ELA-treated MSCs presented enhanced cell viability and decreased apoptosis, which indicated that ELA treatment is a beneficial method of extending MSC’s longevity during H/I injury. In view of this, we analyzed the possible mechanism of action behind ELA.

It is well known that mitochondrial dysfunction is a central event throughout the apoptosis process [36]. Normal MMP is a prerequisite for maintaining mitochondrial oxidative phosphorylation and ATP production [37]. MMP loss occurs prior to the pathological changes in the early stage of apoptosis and is widely regarded as a vital marker for evaluating the early stages of apoptosis [38]. Our data showed that the H/I condition led to MMP depolarization, while ELA could sustain the stability of MMP. Combined with the results obtained from flow cytometry, the early apoptosis rate of MSCs in the ELA group decreased to almost half of that in the H/I group, which suggested that ELA could function in an early stage of MSC apoptosis induced by H/I injury. The gradual dissipation of MMP ultimately reduces ATP synthesis [39]. Consistent with these concepts, ATP synthesis declined severely during H/I injury, but this phenomenon was ameliorated in the ELA group.

The Bcl-2 family members act as crucial factors that influence cell apoptosis [17, 19]. Normal mitochondrial function depends on the integrity of the mitochondrial membrane strictly regulated by Bcl-2 family proteins [40]. The balance of cell death and survival signals in the Bcl-2 family determines the fate of cells. Among which, numerous studies suggest that the Bcl-2/Bax ratio is closely related to cell apoptosis [41]. When pro-apoptotic proteins dominate, such as Bax, the mitochondrial membrane permeability increases, followed by the release of cytochrome c into the cytoplasm, thus activating the protease cascade reaction mediated by the caspase family [42]. It has been proven that Caspase 3 is the critical caspase that executes cell apoptosis [15, 16]. The results displayed that ELA treatment could remarkably increase the Bcl-2 expression but reduce the Bax expression, which suggested that the anti-apoptotic signal Bcl-2 dominated in the ELA group. Besides, the activity of Caspase 3 was inhibited by ELA treatment. Taken together, we presented that ELA treatment could partially affect anti-apoptosis under an H/I state by improving mitochondrial dysfunction.

Intriguingly, ELA had no anti-apoptotic effects on APJ-silenced MSCs, including such aspects as higher apoptosis rate, MMP loss, interrupted ATP synthesis, a decreased Bcl-2/Bax ratio, and active state of Caspase 3. This suggested that ELA protected MSC apoptosis against H/I injury with the help of the APJ receptor. The APJ gene is widely expressed in vast majority of various tissues, including the heart, liver, adipose tissue, and limbs, and it is related to multiple biological processes, such as angiogenesis, energy metabolism, and embryonic development [23, 43, 44]. Our preliminary experiments were conducted to explore the role of ELA at different concentrations to promote the cell proliferation and survival of MSCs in the state of ischemia and hypoxia, among which ELA at the concentration of 5 μM displayed the best function (unpublished data). In this study, we demonstrated that at a concentration of 5 μM, ELA showed optimal affinity with APJ under H/I conditions in MSCs (unpublished data). Whether this affinity is concentration-dependent remains unknown and needs to be verified by future experiments.

It has been well characterized that the ERK1/2 and PI3K/AKT signaling pathways are critical regulators during the apoptosis process [10, 45]. It is important to note the crosstalk between these two signaling axes with mitochondria. The active ERK1/2 signal has been proposed to be a major regulator of anti-survival Bcl-2 and an inhibitor of MMP depolarization [46, 47]. On the other hand, AKT phosphorylation could deprive the pro-apoptotic activity of Bax by maintaining stable mitochondrial membrane permeability [48]. Since the Bcl2/Bax ratio was enhanced in the ELA group with improved MMP depolarization, we further detected the activity of ERK1/2 and PI3K/AKT pathways. Our results showed that both ERK1/2 and AKT phosphorylation levels were reduced within 24 h of H/I exposure in MSCs. However, ELA treatment induced the phosphorylation of ERK1/2 and AKT in MSCs. By contrast, ERK1/2 and AKT phosphorylation could not be detected in APJ-deficient MSCs. Additionally, U0126 blocked the ERK1/2 phosphorylation activated by ELA, and LY294002 reversed the AKT phosphorylation induced by ELA. Therefore, the ERK1/2 and AKT signaling pathways were involved in the anti-apoptotic effects of ELA on MSCs during the H/I state. Moreover, APJ might be one of the upstream molecules of AKT and ERK. Nevertheless, it is necessary to conduct further research to probe the precise and entire downstream factors of ERK1/2 and AKT.

## Conclusion

Our study demonstrated that ELA could promote the anti-apoptosis of MSCs under hypoxic/ischemic conditions. This beneficial property of ELA is relevant to the ameliorated mitochondrial dysfunction and activation of PI3K/AKT and ERK1/2 signaling pathways in an APJ-dependent manner. Our findings revealed that ELA may be considered as a promising cell processing for extending the longevity of MSCs in ischemic myocardium.

## Data Availability

All data generated or analyzed during this study are included in this published article.

## Abbreviations

MSCs: Mesenchymal stem cells
ELA: ELABELA
APJ: Apelin receptor
PI3K: Phosphatidylinositol 3 kinase
p-AKT: Phosphorylated AKT
t-AKT: Total AKT
p-ERK1/2: Phosphorylated ERK1/2
t-ERK1/2: Total ERK1/2
Δψm or MMP: Mitochondrial membrane potential
ATP: Adenosine triphosphate
siRNA: Small interfering RNA
CCK8: Cell counting kit-8
PVDF: Polyvinylidene difluoride
